# Understanding the decision to screen for lung cancer or not: A qualitative analysis

**DOI:** 10.1111/hex.12975

**Published:** 2019-09-27

**Authors:** Claire Burke Draucker, Susan M. Rawl, Emilee Vode, Lisa Carter‐Harris

**Affiliations:** ^1^ Indiana University School of Nursing Indianapolis Indiana; ^2^ Indiana University Simon Cancer Center Indianapolis Indiana; ^3^ Department of Psychiatry & Behavioral Sciences Memorial Sloan Kettering Cancer Center New York New York

**Keywords:** behaviour, long‐term smokers, lung cancer screening, qualitative

## Abstract

**Background:**

Although new screening programmes with low‐dose computed tomography (LDCT) for lung cancer have been implemented throughout the United States, screening uptake remains low and screening‐eligible persons' decisions to screen or not remain poorly understood.

**Objective:**

To describe how current and former long‐term smokers explain their decisions regarding participation in lung cancer screening.

**Design:**

Phone interviews using a semi‐structured interview guide were conducted to ask screening‐eligible persons to describe their decisions regarding screening with LDCT. The interviews were transcribed and analysed with conventional content analytic techniques.

**Setting and participants:**

A subsample of 40 participants (20 who had screened and 20 who had not) were drawn from the sample of a survey study whose participants were recruited by Facebook targeted advertisements.

**Results:**

The sample was divided into the following five groups based on their decisions regarding lung cancer screening participation: Group 1: no intention to be screened, Group 2: no deliberate consideration but somewhat open to being screened, Group 3: deliberate consideration but no definitive decision to be screened, Group 4: intention to be screened and Group 5: had been screened. Reasons for screening participation decisions are described for each group. Across groups, data revealed that screening‐eligible persons have a number of misconceptions regarding LDCT, including that a scan is needed only if one is symptomatic or has not had a chest x‐ray. A physician recommendation was a key influence on decisions to screen.

**Discussion and conclusions:**

Education initiatives aimed at providers and long‐term smokers regarding LDCT is needed. Quality patient/provider communication is most likely to improve screening rates.

## 1 INTRODUCTION

Lung cancer screening with annual low‐dose computed tomography (LDCT) is recommended by the U. S. Preventive Services Task Force (USPSTF) for long‐term current and former smokers.^1^ Lung cancer is the deadliest form of cancer; nearly 1.8 million people including 154 050 Americans are expected to die from lung cancer in 2019.^2^, ^3^ Lung cancer kills more people in the United States than breast, colorectal, pancreatic, and prostate cancers combined.^4^ Most die because they are diagnosed at an advanced stage, and, until recently, an effective screening test did not exist.

LDCT of the chest is promising for the early detection of lung cancer. The National Lung Screening Trial (NLST), the largest randomized controlled trial to date on lung cancer screening, found an approximate 20% relative reduction in lung cancer‐related mortality in high‐risk individuals (age 55‐74, current or former smokers who have quit within the past 15 years, and have at least a 30 pack‐year history of cigarette smoking) screened with annual LDCT compared to chest x‐ray over a 3‐year period.^5^ More recently, the Dutch‐Belgium Lung Cancer Screening [NELSON] Trial also found decreased lung cancer mortality in high‐risk individuals screened with LDCT of the chest compared to chest radiography.^6^ In 2013, the NLST results led to the Grade B recommendation from the US Preventive Services Task Force of lung cancer screening with LDCT for high‐risk individuals (aged 55‐80 years with a minimum 30 pack‐year smoking history who are current or former smokers who quit within the past 15 years).^7^ Subsequently, the Centers for Medicare and Medicaid Services issued a coverage determination to cover lung cancer screening among high‐risk individuals with an upper age limit of 77.^8^

Although new screening programmes with LDCT have been implemented throughout the United States, screening uptake remains low^9^ and persons' decisions to screen or not screen for lung cancer remain poorly understood.^10^, ^11^, ^12^ The purpose of this study was to describe how current and long‐term smokers explain their decisions to participate or not in screening for lung cancer with LCDT.

## 2 PARENT STUDY: IDENTIFYING FACTORS ASSOCIATED WITH LUNG CANCER SCREENING

Data for this study were collected as part of a larger sequential explanatory mixed methods study designed to identify factors associated with lung cancer screening (referred to as the parent study). The study consisted of Phase 1, a quantitative phase, and Phase II, a qualitative phase. Both phases of the parent study were approved by the authors' institutional review board, and all research team members had human subjects training. Although Phase II is the focus of this report, Phase 1 is first briefly described as it provided the context of Phase II.

### 2.1 Phase I: quantitative study

**Figure 1:**
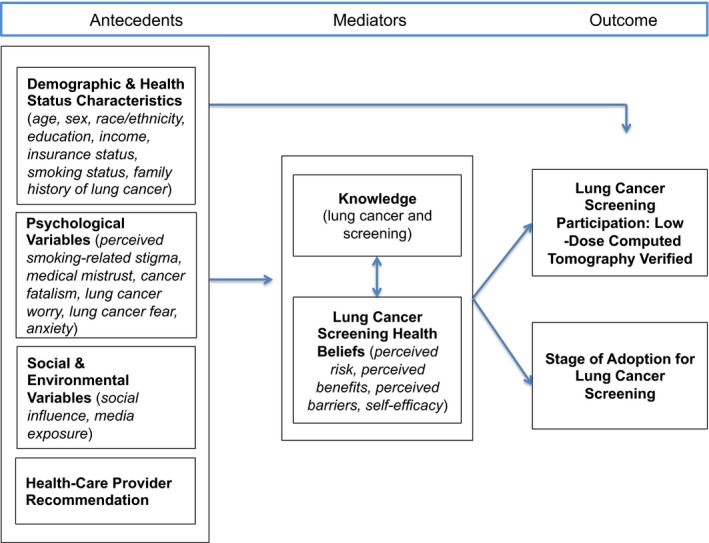
Conceptual model for lung cancer screening participation

The aim of Phase I was to test an explanatory framework for lung cancer screening participation. As seen in Figure 1, the antecedent variables are psychological variables, demographic and health status characteristics, cognitive variables, health‐care provider recommendations (y/n), and social and environmental variables. The mediating variables are lung cancer screening health beliefs, and the outcome variable is lung cancer screening participation (y/n).

A 88‐item self‐report web‐based survey was developed to measure each of the variables in the model. To measure lung cancer screening participation, participants were asked if they had a LDCT scan in the past 12 months to screen for lung cancer. They were given a description of LDCT of the chest and provided a photograph of the scanner so as to distinguish the procedure from a chest x‐ray.

A purposive sample of 515 long‐term current and former smokers in the United States who were eligible for lung cancer screening per USPSTF guidelines were recruited via a nationwide Facebook targeted advertisement. Facebook has the ability to ‘target’ an advertisement by demographics and keywords listed in each individual Facebook user's profile or interest list, which allowed us to purposively sample people who were age 55 years and older and indicated smoking as an interest.

Data were collected using REDCap, a secure web‐based platform. Interested persons completed a screening questionnaire and, if eligible, saw a message that invited them to participate in the study. Embedded in the message was an informed consent document that could be signed electronically. More information about Phase I is presented elsewhere (under review).

### 2.2 Phase II: qualitative study

Phase II was a qualitative study in which telephone interviews were conducted with a sub‐set of the participants in Phase I. Participants in Phase II were asked to explain and expand on some of their responses to items in the Phase I survey.

### 2.3 Research team and training

Phase II was conducted by three senior nurses researchers, a clinical research project manager and six baccalaureate nursing students in an honours programme. The senior researchers included two nurses with expertise in cancer prevention and one with expertise in qualitative methods. The nursing students and the project manager conducted the interviews and did initial coding on the transcripts and the senior researchers further analysed the data to address the study aim.

The qualitative methods expert provided a series of training sessions on qualitative interviewing and basic coding techniques. The training consisted of presentations on qualitative interviewing and coding strategies, role playing exercises to develop interviewing skills, and practice coding sessions. The qualitative expert also reviewed all of the taped interviews and provided ongoing feedback as the interviews were conducted.

### 2.4 Sampling and recruitment

Participants who completed the survey were asked if they would be willing to participate in a follow‐up phone call and 336 expressed a willingness to do so. Selective sampling was used to recruit a subsample of 40 persons from those who agreed to participate in the telephone interviews. Phase II sampling procedures were aimed at ensuring participants whose screening decisions converged with, or diverged from, the Phase I explanatory framework in a variety of ways were interviewed. Therefore, we selected 20 participants who had screened and 20 who had not. Within each group, we included 10 persons whose scores on the psychological and cognitive/health belief measures were hypothesized to be favourable to screening and 10 persons whose scores on these measures were hypothesized to be unfavourable to screening. The creation of the profiles and the selective sampling of participants is described in more detail in Appendix S1. Those who were selected were contacted by email or phone and scheduled for a phone interview.

### 2.5 Data collection

Tailored semi‐structured interview guides were developed for each participant in Phase II based upon whether they had screened or not combined with their scores on the psychological and/or cognitive and health belief factors subscales in Phase I (see Appendix S2). The first question on the survey in Phase I was whether participants had a LDCT to screen for lung cancer in the past 12 months. During the interview, the interviewer confirmed their response. If the participant had screened, they were asked how they had decided to be screened. If they had not screened, they were asked how they decided not to screen. The interviewers then referred to the participants' high or low results on the subscales and asked participants to elaborate on their responses. For example, if a participant had scored low on the medical mistrust subscale, they would be asked to describe interactions with providers that lead to mistrust.

The interviews lasted between 15 and 60 minutes. While some participants were reticent and difficult to engage, most were forthcoming with their thoughts and feelings about lung cancer screening. The interviews were digitally recorded and transcribed verbatim by a member of the research team. Each transcript was given an identification number (001‐040), and no identifying data were included on the transcripts.

### 2.6 Data analysis

Data were analysed using conventional content analysis.^13^ Conventional content analysis is an inductive process that allows codes and categories to emerge from the data rather than a deductive process confirming or refining a pre‐existing theoretical structure.^13^ To address the research aim, we focused primarily on the participants' direct responses to the interviewer's initial query about why they had chosen to be screened for lung cancer or not. While later questioning sometimes provoked more reflective responses about decisions to screen (eg *Did your experience with stigma influence your decision to screen?*), we determined that it was important to first examine participants' spontaneous unprompted explanations of their screening decisions. If, however, participant responses to later questions provided additional clarity about their stated reasons for their screening decisions, these data were brought to bear on the analysis as well.

The nursing students and the project manager were each assigned transcripts to code individually. All data relevant to the participants' stated reasons for being screened or not were extracted and divided into text units (eg words, phrases, sentences or short passages capturing a single thought). They labelled each text unit with a short code that captured its essence. A senior researcher verified each of the codes by re‐examining the transcripts.

The codes were then entered into data display tables.^14^ The tables were constructed by assigning a row to each participant and labelling it with their study ID number. The table had two columns—one displayed each participant's basic demographic information, including their age, gender, ethnicity and smoking status, and one displayed all the codes associated with that participant. The rows were initially divided into two groups according to whether the participant had been screened or not.

The senior researchers met to summarize the information on the data display tables using a process of discussion and consensus. Because the analysis involved a low level of interpretation, analytic disagreements were few and easily resolved by a re‐examination of the data. When examining the interview responses for those who had not been screened, we recognized that they were a heterogeneous group in terms of their screening decisions, which ranged from having made a firm decision not to be screened to having made a firm decision to be screened. We therefore divided the sample into five groups: persons who had decided not to be screened (Group 1), persons who had not deliberately considered being screened but were at least somewhat open to it (Group 2), persons who had deliberately considered being screened but had not made a definitive decision (Group 3), persons who fully intended to be screened but had yet to do so (Group 4) and persons who had been screened at the time of the interview (Group 5).

The codes associated with each group were compared for similarities and differences and grouped into categories. The categories, which represented a variety of reasons that participants in each group decided to be screened or not, were validated with a re‐examination of the transcripts. The research team determined that the 40 interviews yielded ample data to provide a robust description of how current and long‐term smokers explain their decisions to participate or not in screening for lung cancer with LCDT. A narrative description of each category was prepared and reviewed by the research team.

## 3 RESULTS

### 3.1 Sample

Forty persons participated in the interviews, but data from one person were lost due to recording equipment failure. One participant had indicated on her survey that she had screened but denied this during the interview and was therefore considered a non‐screener for this analysis. No participants reported having a lung scan between the survey and the interview. The final sample thus included 39 participants: 18 persons who had screened and 21 persons who had not screened. Participants hailed from 20 states representing all geographical regions of the United States.

Of the final sample of 39, 26 were women and 13 were men. Participants ranged in age from 55 to 70, with an average age of 62 years. Thirty‐five were Caucasian, one was African American, one was American Indian/Alaskan Native, and two were mixed race (ie African American and Native Hawaiian, Caucasian and American Indian). Twelve were divorced, 10 were married, seven were never married, five were living with a partner, and five were widowed. Twenty‐eight were not working for pay, six were working full‐time, and five were working part‐time. Eleven had graduated from college, 21 had completed some college, six had graduated from high school, and one had not graduated from high school. Eight had an annual income of >$50k, 18 between $25K and $50k, and 13 < $25k. Twenty‐one participants were former smokers and 18 currently smoked. Information about earlier screening experiences other than the LDCT or illness history was not collected in the Phase I survey.

### 3.2 Decisions regarding participating in lung cancer screening

In response to the interviewers' initial questions about decisions regarding lung cancer screening participation, most participants provided clear reasons why they had screened or alternatively why they had not. The reasons each of the five groups, identified above, provided for their screening decisions are described below. The description of the groups are ordered from those most resistant to being screened (Group 1) to those who had been screened (Group 5). For each verbatim quote, the participant's study identification number (ID) is included in parentheses.

#### 3.2.1 Group 1: no intention to be screened

Some participants had no intention to be screened because they felt it was unnecessary. Most of these participants were fairly adamant that a lung scan would be a ‘waste of time’, ‘not worth the time or effort’ or a needless expense. They indicated that their physician either had not mentioned a lung scan or recommended against it. A 65‐year‐old man who currently smoked (ID: 018) stated, ‘I firmly believe that if he [his physician] felt it [the lung scan] was necessary, he would have recommended it’.

Several participants thought a lung scan was unnecessary because they did not have symptoms associated with lung cancer. A 57‐year‐old woman who currently smoked (ID: 032) said, ‘I don't have any symptoms or any reason to go [get a lung scan…]’. This participant and others assumed they would surely have symptoms if they had lung cancer.

Others were convinced that a lung scan was redundant if they had had chest x‐rays or if their physician routinely ‘listened to’ their heart and lungs. A 68‐year‐old man who formerly smoked and was an x‐ray technologist (ID: 012) said, ‘I feel if a chest x‐ray doesn't show it, what good is a CT? If you're going to get it [lung cancer], it's going to show up on a chest x‐ray eventually, so to waste somebody's time when someone else needs a CT…’

#### 3.2.2 Group 2: no deliberate consideration but somewhat open to being screened

Some participants who had not had a lung scan had not deliberately considered being screened. These participants, unlike those in Group 1, did not maintain it was unnecessary but they had not thought much about it. A 58‐year‐old woman who currently smoked (ID: 034) said, ‘I just never considered it. I've always been pretty healthy…’

Like Group 1, participants in Group 2 indicated that their physicians had not discussed screening. Moreover, participants in this group knew little about lung scans. A 55‐year‐old woman who formerly smoked (ID: 015) said, ‘If you had knowledge of exactly what it [lung scan] is … I think more people would have it done’.

Unlike participants in Group 1, however, those in Group 2 expressed some openness to being screened, although their commitment was typically tepid. Some indicated they would consider having a lung scan under certain conditions, such as if a physician recommended it or if they started to feel badly or develop symptoms. A 62‐year‐old woman who formerly smoked (ID: 027) said, ‘Yes, that's [screening] a possibility because maybe if I was going to the same person [provider] all of the time and I had been recommended on maybe more than one occasion I might consider doing it’.

#### 3.2.3 Group 3: deliberate consideration but no definitive decision to be screened

Some participants who had not had a lung scan had considered being screened in an intentional way. Unlike Group 2, they were familiar with lung scans and having one had been on their minds. A 55‐year‐old woman who formerly smoked (ID: 020) said, ‘I thought about it many times…’

Participants in Group 3 revealed a number of reasons why they had not been screened. Like Groups 1 and 2, they indicated that their physicians had not recommended a lung scan. A 70‐year‐old woman who currently smoked (ID: 008) said, ‘It [lung scan] hadn't been recommended to me, [but] I've thought about it’. For some, the decision to get a lung scan hinged on their physician's recommendation. The 55‐year‐old woman mentioned above (ID: 020) who had thought of having a lung scan many times said that she would ‘absolutely want’ a scan if her physician recommended it to her.

A few participants in this group stated they had not had the time to get screened, whereas others were deterred by a variety of other factors. The cost of the scan was a concern for some of the participants who said they would have the scan if it was free or covered by insurance. Other participants wanted to be screened but were reluctant to get a scan because they feared the results. A 55‐year‐old woman who formerly smoked (ID: 014) said, ‘I am going to go [to get a lung scan] but right now I haven't gone. Eventually I will go. I'm trying to work up the courage’. Other reasons participants put off screening included having other more pressing health issues and having a serious ill family member who required their attention.

#### 3.2.4 Group 4: intention to be screened

Unlike the participants in Groups 3 who considered a lung scan but had not decided definitively to have one, participants in Group 4 had made a fairly firm decision to be screened. These participants indicated that they had planned to get screened and were committed to following through. A 55‐year‐old woman who formerly smoked (ID: 017) said, ‘Yes, I have considered it [having a lung scan] being that I do have COPD and I have been a smoker for over 40 years. So, yes, I plan on being screened as soon as possible’.

These participants discussed why they delayed getting a lung scan in light of the fact that they were determined to be screened. In some instances, participants were hindered by a physician who was not supportive of the lung scan. A 62‐year‐old woman who formerly smoked (ID: 017) decided to have a lung scan done ‘as a baseline’ and went to ‘the x‐ray place’ to have it done. However, she was told to get a ‘written order’ from her physician for the scan. The participant indicated that her physician refused to provide the order because he believed the scan was unnecessary as she was asymptomatic. A 63‐year‐old woman who currently smoked (ID: 021) had decided to have a lung scan, along with a number of other screening procedures, when she received Medicare in a few months following the interview.

#### 3.2.5 Group 5: had been screened

All persons who had had a lung scan at the time of the interview clearly articulated at least one reason why they chose to be screened and several provided more than one reason. The most common reasons included experiencing pulmonary problems, receiving a recommendation from a physician and valuing early detection.

##### Experiencing pulmonary problems

Some participants indicated that they had a lung scan because they had a pulmonary illness such as chronic obstructive pulmonary disease (COPD), emphysema, collapsed lung, pulmonary hypertension, or scleroderma or were experiencing ‘breathing problems’, shortness of breath or a cough. A 64‐year‐old woman who formerly smoked (ID: 001) said, ‘I was screened because I do have Stage 4 COPD, so of course they did test for cancer’. Some of these participants indicated that the scan was a necessity rather than a choice.

##### Receiving a recommendation from a physician

Some participants indicated that they had been screened on the recommendation of their physician. Some physicians had recommended the scan because the participant was having some pulmonary problems as described above, but others recommended it in the absence of known problems due to the participants' smoking history. Participants typically did not question the advisability of a lung scan when recommended by their physicians. A 69‐year‐old woman who currently smoked (ID: 023) said, ‘My doctor suggested it. Of course, being a smoker I said, well, that's a good idea. I had no problem with it…’

##### Valuing early detection

Some participants indicated that they had a lung scan because they valued preventing or detecting medical problems early. They indicated that they had a lung scan because they wanted to ‘stay on top of [my] health’, felt ‘early detection is best’ and ‘wanted to catch it [cancer] early’. Some compared the advisability of having a lung scan to that of other routine screening procedures such as mammography or colonoscopy. A 64‐year‐old woman who formerly smoked (ID: 001) said, ‘I try to stay on top of various medical issues, just so that if there is something going on, I can catch it as early as possible’. Some of these participants, rather than their health‐care providers, initiated the lung scan. A 56‐year‐old man who formerly smoked (ID: 024) learned about the lung scan because his hospital was running a ‘special’ that was widely advertised. He said, ‘I decided to get screened on my own and when I was talking to one of my doctors about it, they said it was a great idea. So I went ahead and did it’.

##### Other reasons

A few participants cited other reasons for having a lung scan. Some indicated that they had a lung scan because it was covered by their insurance as a ‘preventative tool’. Others indicated they had a lung scan because it was required by Medicaid or was part of a research study. One had a scan because of a family history of lung disease.

## 4 DISCUSSION

Consistent with our prior work,^12^ participants' decisions to screen for lung cancer ranged from a firm decision not to screen because they believed it was unnecessary and a waste of time to a decision to screen based on a firm conviction that screening was essential to their health and well‐being. Factors that were most likely to promote screening included perceived physician endorsement and experiencing pulmonary symptoms. Factors that impeded decisions to screen included the belief that other tests detect cancer, cost of the scan, fear of the results and other life stressors.

As a result of the findings, the team determine that the groups identified in this analysis aligned with precaution adoption process model (PAPM)^15^ and each group could be placed to be in one of the stages outlined in the model. The PAPM focuses on how people make decisions to take action or not regarding health issues and how they translate these decisions into actions. The seven stages in the model are as follows: stage 1: unaware of issue, stage 2: unengaged by issue, stage 3: undecided about acting, stage 4: decided not to act, stage 5: decided to act, stage 6: acting and stage 7: maintenance. People who are undecided about taking action (stage 3) can either stay in that stage or decide not to act (stage 4) or decide to act (stage 5). No persons who participated in our interviews would have been in Stage 1 because all would have been aware of LDCT scans for lung cancer due to their participation in the study. However, participants in our Group 2 could be considered to be in Stage 2 of the PAPM as they were unengaged in thinking about screening. Participants in our Group 3 could be considered to be in Stage 3 of the PAPM because they were engaged in making a decision but undecided about whether or not to have a scan. Participants in our Group 1 could be considered to be in Stage 4 of the PAPM as they had made a deliberate decision not to be screened. Participants in our Group 4 could be considered to be in Stage 5 of the PAPM because they had decided to be screened, and participants in our Group 5 could be considered to in Stage 6 of the PAPM because they had followed through with screening. Because the recommendation for annual lung cancer screening with LDCT of the chest is fairly recent, no one in our sample had had a series of lung scans for maintenance, which defines Stage 7 of the PAPM.

Regardless of the current lung cancer screening status of participants, recommendations of physicians were paramount to the decision participants made about screening. Many indicated that they screened without hesitation on the advice of their physicians, whereas no participants went against the advice of physicians who had encouraged them to be screened. Most commonly, participants who had not been screened indicated their physicians had not mentioned lung cancer screening, had not strongly recommended it or had recommended against it. These findings are consistent with our previous studies that found that a provider recommendation is a strong predictor of lung cancer screening participation^16^, ^17^, ^18^ and with studies on other types of cancer screening that found that a provider recommendation is the best predictor of breast and colorectal cancer screening.^17^, ^19^, ^20^, ^21^

The results also revealed that several participants held inaccurate beliefs that influenced their decisions about lung cancer screening participation. Our findings support similar research confirming lack of knowledge about the concept of screening.^12^, ^22^, ^23^ A common misconception was that those with lung cancer are symptomatic and thus screening was unnecessary in the absence of symptoms. Another misconception was that routine examinations and chest x‐rays would reveal lung cancer thereby rendering a LDCT redundant. These two misconceptions discouraged screening and thus could impede detection of lung cancer at early, more treatable stages. Moreover, several participants were convinced that lung cancer screening would be very expensive and not covered by insurance. Although this concern was found in with our prior studies examining screening barriers,^16^, ^17^ lung cancer screening is a Grade B recommendation by the USPSTF with zero copayment for Medicare beneficiaries.

## 5 LIMITATIONS

The findings should be considered in the context of several study limitations. Determination of screening status was based on self‐report, and a few participants needed to be reminded a lung scan was different than a chest x‐ray. However, all participants had been provided a picture of a LDCT scan as well as a description of the scan and its purpose. Another limitation was that prior participation in Phase I may have influenced the findings of Phase II. Some participants may have discussed a willingness or intent to screen during their interviews because the survey had implied that screening was important. Moreover, some may have engaged in behaviours following the survey that they might not have otherwise, such as learning more about screening or discussing screening with family, friends, or their health‐care providers. However, during the interviews, no participants revealed that the survey influenced their subsequent behaviours or their decisions about screening.

Facebook recruitment may have also limited the findings. While 69% of the adult population use Facebook,^24^ it is unclear if they differ from adults who do not use Facebook in ways that might influence screening decisions (eg more exposure to information about the health hazards of smoking or the availability of LDCT).

Another study limitation was the lack of ethnic diversity in the sample. Only four participants (10% of the sample) were from minority groups. This may have stemmed from the well‐documented phenomenon that minority persons, especially African Americans, are less likely participate in research due to mistrust.^25^, ^26^ Our recruitment methods might have also contributed to the lack of diversity. While the proportion of White and Black adults in the United States who use Facebook is equal (70% for both groups),^24^ some methodologists have suggested that targeted paid advertisements are not always successful in encouraging all groups to engage in research and the use of other strategies, such as direct communication with existing Facebook groups that address issues of importance to the minority group to be recruited and networking with page administrators who can promote the study, may be needed.^27^ We also recognize that there were twice as many women as men in the qualitative sample and this could have had an impact on results. However, our prior work on the role of gender in lung cancer screening behaviour has not revealed any gender‐related differences.^28^

In addition, in Phase I, we did not collect data on some factors that might have influenced screening decisions, such as prior cancer screening and prior cancer experiences, and this information could have added to the Phase II analysis. Moreover, our analysis did not allow us make definitive conclusions about whether persons who were current or former smokers differed in their reasons to screen or not. However, there was no manifest indication that smoking status influenced decisions to screen. For example, former smokers did not indicate they avoided screening because they were not at risk for lung cancer and current smokers did not indicate they avoided screening because the results might be a cause for them to stop smoking. In addition, because our interviews occurred at a single time point, we could place participants in one of the five groups but could not account for dynamic changes in screening over time, such as those outlined in the PAPM^15^ model. For example, we recognize that behavioural intentions are not the same as actual behaviour change^29^ and our findings do not explain how participants move from intending to screen to actually getting screened.

To address the limitations of this study, we suggest a study in which screening‐eligible persons are interviewed at several time points to capture the dynamic unfolding of screening participation. We also recommend recruiting an ethnically diverse sample and exploring the influence of a number of factors, such as smoking status and sociodemographic background, on screening decisions. For example, while determining the effects of demographic factors, such as age, gender, race and income, on screening decisions was beyond the scope of this study, future research with larger and more diverse samples could explore such influences.

## 6 CLINICAL IMPLICATIONS

Our study confirms that education regarding lung cancer screening should target both patients and providers and should be widely implemented. Similar to findings by Raz and colleagues,^30^ our findings suggest that such efforts should clarify the difference between screening for lung cancer to detect it early in the absence of symptoms and diagnostic testing to identify aetiology when symptoms are present. Education initiatives should stress that LDCT is the only effective and approved test to screen for lung cancer and dispel the misconception that a chest x‐ray is an effective screening test. Moreover, all providers need to be aware of national guidelines and practice recommendations and the evidence that supports screening with LDCT. Targeted provider education should be offered through multiple venues (ie professional organizations, public service announcements, clinical pearls [tips for clinicians to apply in clinical practice], continuing education offerings). Our findings also suggest that high‐quality patient‐provider communication about lung cancer screening should occur regularly. Given that our participants' reasons for not screening were varied and in some instances evolving, opportunities to discuss their hesitancy to screen with a provider would likely improve screening rates.

## 7 SUMMARY

During phone interviews, screening‐eligible persons were asked to explain their decisions about participation in screening with LDCT. Some revealed they decided to screen because they were firmly convinced of its benefit, some revealed they decided not to screen because they were certain it was unnecessary, and others discussed being open to screening, thinking about screening or intending to screen. Although a variety of factors influenced their decisions, including several misconceptions about LDCT, their health‐care providers' role in discussing or endorsing screening was paramount. The findings indicate that education regarding lung cancer screening is needed for screening‐eligible persons and their providers and that high‐quality patient‐provider communication regarding screening is most likely to improve screening rates.

## CONFLICT OF INTEREST

None.

## Data Availability

Data consisted of transcripts of semi‐structured interviews that might contain information that could identify participants and thus will not be available.
