# Supplementary material for: Understanding the decision to screen for lung cancer or not: A qualitative analysis
Source: Health Expect. 2019 Sep 27;22(6):1314–21. doi: 10.1111/hex.12975 (PMC6882261; doi:10.1111/hex.12975)
Supplement: Supplementary file 1 [file HEX-22-1314-s001.docx]

Appendix S1

Screening Protocol for Phase II

**Procedures:** Quantitative data collected in Phase I were analyzed to create individual participant profiles in order to selectively sample participants for telephone interviews about their screening choices. The goal of sampling for Phase II was to select information-rich cases to obtain additional information about participants’ screening choices. Because we were interested in understanding factors that influence screening-eligible individuals to screen or not screen for lung cancer, we selected an equal number of cases from these two groups (screeners, n=20; non-screeners, n=20) based on their responses to the self-reported Lung Cancer Screening Participation item (Y/N). To allow us to explore the context and mechanisms of varied pathways to screening and non-screening, we selectively sampled cases with varied profiles of factors. To create the profiles, we focused on (a) psychological factors (perceived stigma, mistrust, fatalism, worry, and fear) and (b) cognitive and health belief factors (knowledge, perceived risk, perceived benefits, perceived barriers, and self-efficacy) thought to be favorable or unfavorable to screening. Cutoff points for the individual measures were used to create profiles that presented in the Table 1 below. For example, an individual with a total score of 13 on the Cataldo Lung Cancer Stigma Smoking Subscale,^31^ 20 on the Patient Trust in the Medical Profession Scale,^32^ and 18 on the Lung Cancer Fear Scale^33^ would be classified as having a profile that is Unfavorable to Screening. Feeling blamed for being a smoker, mistrusting one’s healthcare provider, and feeling threatened by what it would mean to be diagnosed with lung cancer are likely to dissuade lung cancer screening.

| **Table 1: Procedures for Determining Profile Patterns** | |
| --- | --- |
| **Profile (*Definition*)** | **How to Calculate Profile** |
| Favorable Psychological Factors  (*Pattern of psychological variables favorable to screening*) | If scores on 3 of the 5 scales:  1. Low stigma: Cataldo Lung Cancer Stigma Smoking Subscale^31^ < 10  2. Low mistrust: Patient Trust in the Medical Profession Scale^32^ < 17  3. Low fatalism: Revised Powe Fatalism Inventory^34^ < 5  4. Low worry: Lung Cancer Worry Scale^35^ < 6  5. Low fear: Lung Cancer Fear Scale^33^< 16 |
| Unfavorable Psychological Factors  (*Pattern of psychological variables unfavorable to screening*) | If scores on 3 of the 5 scales:  1. High stigma: Cataldo Lung Cancer Stigma Smoking Subscale^31^ > 11  2. High mistrust: Patient Trust in the Medical Profession Scale^32^ > 18  3. High fatalism: Revised Powe Fatalism Inventory^34^ > 6  4. High worry: Lung Cancer Worry Scale^35^ > 7  5. High fear: Lung Cancer Fear Scale^33^ > 17 |
| Favorable Cognitive and Health Beliefs  (*Pattern of cognitive and health beliefs favorable to screening*) | If scores on 3 of the 5 scales:  1. High knowledge: Knowledge: Lung Cancer and Lung Cancer Screening Scale^*^ > 4  2. High risk: Perceived Risk of Lung Cancer Scale^16^ > 13  3. High benefits: Perceived Benefits of Lung Cancer Screening Scale^16^> 13  4. Low barriers: Perceived Barriers to Lung Cancer Screening Scale^16^ < 34  5. High self-efficacy: Self-Efficacy for Lung Cancer Screening Scale^16^ > 19 |
| Unfavorable Cognitive and Health Beliefs  (*Pattern of cognitive and health beliefs unfavorable to screening*) | If scores on 3 of the 5 scales:  1. Low knowledge: Knowledge: Lung Cancer and Lung Cancer Screening Scale^*^ < 3  2. Low risk: Perceived Risk of Lung Cancer Scale^16^ < 12  3. Low benefits: Perceived Benefits of Lung Cancer Screening Scale^16^ < 12  4. High barriers: Perceived Barriers to Lung Cancer Screening Scale^16^  > 35  5. Low self-efficacy: Self-Efficacy for Lung Cancer Screening Scale^16^ < 18 |

*^* Investigator-developed measure^*

As seen in Table 2, these profile patterns were used to identify eight diverse sub-groups of five cases with characteristics consistent with or divergent from the conceptual model. For example, participants in Cell 1 were individuals who had screened and who had psychological and cognitive and health belief profiles favorable to screening, whereas participants in Cell 7 were individuals who had not screened and who had favorable cognitive and health belief profiles but unfavorable psychological profiles.

| **Table 2: Sampling Plan** | | | | |
| --- | --- | --- | --- | --- |
|  | **Favorable Psychological Factors** | | **Unfavorable Psychological Factors** | |
| Screeners | Cell 1 (*n* = 5)  Favorable cognitive and health beliefs | Cell 2 (*n* = 5)  Unfavorable cognitive and health beliefs | Cell 3 (*n* = 5)  Favorable cognitive and health beliefs | Cell 4 (*n* = 5)  Unfavorable cognitive and health beliefs |
| Non-screeners | Cell 5 (*n* = 5)  Favorable cognitive and health beliefs | Cell 6 (*n* = 5)  Unfavorable cognitive and health beliefs | Cell 7 (*n* = 5)  Favorable cognitive and health beliefs | Cell 8 (*n* = 5)  Unfavorable cognitive and health beliefs |
| Note: Shaded cells represent cases whose profiles are **consistent** with the conceptual model; white cells represent cases that **diverge** from the conceptual model | | | | |

All the participants from the surveys were placed in one of the cells. Five participants from each cell were randomly selected and were contacted and invited to participate in the telephone interview. If participants declined an invitation to participate or could not be reached, another participant from the same cell was randomly selected to replace those who were unavailable. This process was continued until 40 interviews were conducted with 5 participants in each cell.

References:

[31] Cataldo J, Jahan TM, Pongquan VL, et al. Measuring stigma in people with lung cancer: psychometric testing of the cataldo lung cancer stigma scale. Oncol Nurs Forum. 2011;38(1):E46‐E54.

[32] Dugan E, Trachtenberg F, Hall MA. Development of abbreviated measures to assess patient trust in a physician, a health insurer, and the medical profession. BMC Health Serv Res. 2005;5:64.

[33] Champion VL, Skinner CS, Menon U, et al. A breast cancer fear scale: psychometric development. J Health Psychol. 2004;9(6):753‐762.

[34] Mayo RM, Ureda JR, Parker VG. Importance of fatalism in understanding mammography screening in rural elderly women. J Women Aging. 2001;13(1):57‐72.

[35] Hay JL, Buckley TR, Ostroff JS. The role of cancer worry in cancer screening: a theoretical and empirical review of the literature. Psycho‐Oncol. 2005;14(7):517‐534.
