# Supplementary material for: Understanding the decision to screen for lung cancer or not: A qualitative analysis
Source: Health Expect. 2019 Sep 27;22(6):1314–21. doi: 10.1111/hex.12975 (PMC6882261; doi:10.1111/hex.12975)
Supplement: Supplementary file 2 [file HEX-22-1314-s002.docx]

**Appendix S2. Example Interview Guide Tailored to Participant Survey Responses**

This is an example interview guide for a person who did not screen and who scored: (1) high on stigma, mistrust, fatalism, perceived benefits of lung cancer screening, perceived barriers to lung cancer screening, and self-efficacy for lung cancer screening; and (2) low on lung cancer fear, lung cancer worry, knowledge of lung cancer and screening, and perceived risk for lung cancer.

*_____________________________________________________________________*

**Insert Participant Information here**

*(i.e.: age, gender, race, smoking status, screening status, relationship status, working status, and their scores for each variable.)*

*_____________________________________________________________________*

*Thank you for agreeing to speak with me today. I appreciate your time. As you know, I am going to ask you more about some of the responses you gave on the survey you completed this summer. We really value hearing about your thoughts, experiences, and opinions. However, if there are any questions you prefer not to answer, that is fine – just let me know. Also, we can stop any time you like. Some of the questions may seem repetitious but we want to explore you thoughts about many different factors related to lung cancer screening…*

*Do you have any questions for me? I do want to remind you that this interview will be recorded so we catch all that you say.*

**Your Decision (not to be screened)**

| 1. *I understand that you have not been screened for lung cancer. Is this something that has been recommended to you or that you have considered? [if so] can you tell me about your decision not to screen…* |
| --- |

**Stigma (High)**

| 1. *You indicated that you often feel blamed for being a smoker. Tell me more about that. Give me an example of when that happened.* 2. Have you ever felt that way when interacting with your doctor? [if yes]: Tell me about an interaction you have had with your doctor when you felt blamed because you smoked. 3. *Did any experiences of being blamed influence your decision to be screened [not to be screened]. If so, tell me more about that.* |
| --- |

**Mistrust (High)**

| 1. *You indicated that you don’t completely trust your healthcare provider. Tell me more about that.* 2. *Can you tell me about a particular time when you felt a good deal of mistrust of your provider? Can you tell me about some of the things the provider did that caused you to mistrust him/her?* 3. *How did those experiences influence your decision not to be screened?* |
| --- |

**Fatalism (High)**

| 1. *You indicated that you believe if you are meant to be diagnosed with lung cancer, it will happen no matter what you do. Tell me more about that that belief.* 2. *Have you had any particular life experiences that led you to that belief?* 3. *How did this belief influence your decision not to be screened?* |
| --- |

**Fear (Low)**

| 1. *You indicated that the thought of lung cancer does not scare you. Tell me more about that.* 2. *How did your lack of fear influence your decision not to be screened?* |
| --- |

**Worry (Low)**

| 1. *You indicated that the thought of lung cancer is not something that worries you. Tell me more about that.* 2. *How did your lack of worry influence your decision not to be screened?* |
| --- |

**Knowledge (People with LOW Knowledge)**

| 1. *Your survey indicates that there is information about lung cancer that you may not know about. Tell me how you get information about lung cancer and lung cancer screening.* 2. *How did the information you do have about lung cancer and lung cancer screening influence you decision not be be screened?* |
| --- |

**Perceived Risk of Lung Cancer (Low)**

| 1. *Your survey indicates that you think your risk of getting lung cancer one day is low. Tell me more about that.* 2. *How did that belief influence your decision not to be screened?* |
| --- |

**Perceived Benefits of Lung Cancer (High)**

| 1. *Your survey indicated that you think getting a lung scan to screen for lung cancer would benefit you. Tell me more about that.* 2. *How did that belief influence your decision not to be screened?* |
| --- |

**Perceived Barriers to Lung Cancer Screening (High)**

| 1. *Your survey indicated that there are a number of reasons you might put off having a lung scan. Some of these reasons were [x, y, and z – fill in from survey]. We call these reasons barriers. Tell me more about these barriers. [probe for each barrier].* 2. *Tell me how these barriers influenced your decision not be to screened. Which barrier would you consider the most important – that is, gets in the way of you being screened the most?* |
| --- |

**Self-Efficacy for Lung Cancer Screening (High)**

| 1. *You indicated on your survey that you are confident about getting a lung scan done. Tell me more about that.* 2. *Despite feeling confident, you decided not to be screened. Tell me more about that.* |
| --- |

**Ending Questions**

| 1. *Thanks for the information you shared. Keeping in mind that our goal is to better understand what influences people’s decisions about lung cancer screening, is there anything you can add?* 2. *Is there anything I should have asked and didn’t?* |
| --- |
